# Identification and characterization of the α-CA in the outer membrane vesicles produced by *Helicobacter pylori*

**DOI:** 10.1080/14756366.2018.1539716

**Published:** 2019-01-07

**Authors:** Maurizio Ronci, Sonia Del Prete, Valentina Puca, Simone Carradori, Vincenzo Carginale, Raffaella Muraro, Gabriella Mincione, Antonio Aceto, Francesca Sisto, Claudiu T. Supuran, Rossella Grande, Clemente Capasso

**Affiliations:** a CeSI-MeT Centro Scienze dell’Invecchiamento e Medicina Traslazionale, Center of Aging Sciences and Translational Medicine (CeSi-Met), Chieti, Italy;; b Department of Medical, Oral, and Biotechnological Sciences, University G. d’Annunzio of Chieti-Pescara, Chieti, Italy;; c CNR, Istituto di Bioscienze e Biorisorse, Napoli, Italy;; d Department of Medicine and Aging Science, G. d’Annunzio of Chieti-Pescara, Chieti, Italy;; e Department of Pharmacy, University “G. d’Annunzio” of Chieti-Pescara, Chieti, Italy;; f Dipartimento di Scienze Biomediche, Chirurgiche ed Odontoiatriche, University of Milan, Milan, Italy;; g NEUROFARBA Department, Sezione di Scienze Farmaceutiche e Nutraceutiche, Università degli Studi di Firenze, Sesto, Italy

**Keywords:** Carbonic anydrases, *Helicobacter pylori*, outer membrane vesicles (OMVs), biofilm, protonography, mass spectrometry

## Abstract

The genome of *Helicobacter pylori* encodes for carbonic anhydrases (CAs, EC 4.2.1.1) belonging to the α- and β-CA classes, which together with urease, have a pivotal role in the acid acclimation of the microorganism within the human stomach. Recently, in the exoproteome of *H. pylori,* a CA with no indication of the corresponding class was identified. Here, using the protonography and the mass spectrometry, a CA belonging to the α-class was detected in the outer membrane vesicles (OMVs) generated by planktonic and biofilm phenotypes of four *H. pylori* strains. The amount of this metalloenzyme was higher in the planktonic OMVs (pOMVs) than in the biofilm OMVs (bOMVs). Furthermore, the content of α-CA increases over time in the pOMVs. The identification of the α-CA in pOMVs and bOMVs might shed new light on the role of this enzyme in the colonization, survival, persistence, and pathogenesis of *H. pylori*.

## 1. Introduction

In prokaryotes, the existence of genes encoding carbonic anhydrases (CAs, EC 4.2.1.1) belonging to the α-, β- and γ-classes suggests that these enzymes play an important role in the prokaryotic physiology^1–6^. CAs, in fact, are metalloenzymes catalyzing a common reaction in all life domains: the carbon dioxide hydration to bicarbonate and protons (CO_2_ + H_2_O ⇔ HCO_3_^-^ + H^+^)^5^^,^^7^. In the last years, CAs were investigated in detail in pathogenic bacteria since it has been demonstrated that in many microorganisms they are essential for the life cycle of microbes^1^^,^^8–13^. *Helicobacter pylori* is the causative agent of gastritis, peptic, and duodenal ulcer as well as MALT lymphoma and gastric cancer^1^^,^^14^^,^^15^. The persistence of *H. pylori* in hostile niches is due to an extraordinary ability to adapt itself to different environmental conditions. Two different CAs are encoded by the genome of *H. pylori*: the periplasmic α-CA (hpαCA) and the cytoplasmic β-CA (hpβCA). The bacterial expression of the two CAs is induced under acidic conditions by the two-component system (ArsRS), which is an acid-responsive signaling system^16^. The occurrence and significance of the two CAs and their joint activities with urease have been widely studied to maintain the *H. pylori* periplasmic and cytoplasmic pH close to neutrality in the highly acidic gastric environment. *Helicobacter pylori* buffers its periplasm by means of CO_2_/HCO_3_^−^ and NH_3_/NH_4_^+^ couples produced by the reactions catalyzed by urease and the α- and β-CAs^17^. The gene deletion or inhibition of the *H. pylori* CAs determined a reduced stomach and duodenum colonization *in vivo* limiting the bacterial survival within the highly acid environment^18^^,^^19^. It has also been proved that the absence of hpCAs activity decreased the bacterial membrane integrity^20^. Thus, hpCAs have been proposed as innovative therapeutic targets for the treatment of patients infected with drug-resistant *H. pylori* strains, and several selective inhibitors have been identified so far^21–26^. In addition to the CA/urease system, *H. pylori* possess the capability of producing biofilm for its efficient colonization in the host^27^^,^^28^. The biofilm is a complex tridimensional structure composed of microbial cells immersed in a matrix of extracellular polymeric substances (EPS) consisting of proteins, lipids, carbohydrates, and nucleic acids^29^. Current studies report that the EPS matrix of *H. pylori* biofilm is constituted by proteomannans, LPS-related structures, extracellular DNA (eDNA), proteins, and outer membrane vesicles (OMVs)^30^^,^^31^. The OMVs are spherical bilayered structures of 20–250 nm in diameter released by the microorganisms during their growth both *in vitro* and *in vivo*^32^. The formation of OMVs is due to a protrusion of the outer membrane of the Gram-negative bacterial cell wall that, during its formation, incorporates a variety of macromolecules of periplasmic nature^33^. The produced OMVs are involved in several mechanisms such as pathogenesis, cell-cell communication, biofilm formation, horizontal gene transfer, and nutrient acquisition^32^. The OMVs generated by *H. pylori* have a key role in the microorganism biofilm formation, and in particular, the associated eDNA seems to have a role in “bridging” OMV–OMV and OMV–cell interactions^34^^,^^35^. In a recent study, Snider et al. analyzed the exoproteome of *H. pylori* in different growth phases in the planktonic phenotype^36^. The authors identified 74 proteins selectively released in the extracellular environment by OMVs production. Among them, the authors detected a CA in the later phase of growth, however, no indication of the family was provided. Here, we identify and characterize for the first time an α-CA in the OMVs generated over time from *H. pylori* both in its planktonic and biofilm phenotypes.

## 2. Material and methods

### 2.1. Chemistry

All the chemicals were of the highest purity available, from Sigma-Aldrich (Milan, Italy).

### 2.2. Bacterial strains and media

In the present study, four *H. pylori* strains were used: the reference strain *H. pylori* ATCC43504/NCTC11637 (*Hp* NCTC) and 3 clinical strains, *H. pylori* 190 (*Hp* 190), *H. pylori* F1(*Hp* F1) and *H. pylori* F4 (*Hp* F4) characterized by a different antimicrobial susceptibility pattern as reported in Table 1 on the basis of EUCAST (European Committee on Antimicrobial Susceptibility Testing) breakpoints. The stocks were stored at −80 °C before being thawed at room temperature, plated on Chocolate Agar (CA; Oxoid Limited, Hampshire, UK), supplemented with 1% (v/v) of IsoVitaleX (Becton Dickinson, Franklin Lakes, NJ, USA) and 10% (v/v) of defibrinated horse sterile blood (Oxoid Ltd), and finally incubated at 37 °C for 3 days in a microaerophilic atmosphere (Campy Pak Jar; Oxoid Ltd; Drumm and Sherman, 1989). The antimicrobial susceptibility testing versus *H. pylori* F1 and *H. pylori* F4 was performed following the methodology used by Sisto et al^37^.

**Table 1.** Antimicrobial susceptibility pattern of *H. pylori*.

Bacterial strains	Clinical isolation	Antimicrobial susceptibility	References
*H. pylori* ATCC43504/NCTC11637	Human gastric antrum	MNZ^R^	Sisto et al.^37^
*H. pylori* 190	Gastritis	CLA^S^, MNZ^S^	Sisto et al.^37^
*H. pylori* F1	Gastritis	CLA^R^	This study
*H. pylori* F4	Gastritis/ulcer	CLA^R^, MNZ^R^	This study

MNZ: Metronidazole; CLA: Clarithromycin; R: Resistant; S: Sensitive.

### 2.3. Biofilm formation assay and the isolation of OMVs

The strains were cultivated in Brucella Broth (BB; Oxoid Ltd, Hampshire, UK), supplemented with 2% (w/v) of fetal calf serum (Sigma Aldrich, St. Louis, MO, USA) and 0.3% (w/v) of glucose^30^ and the biofilms were developed as previously reported^34^. The formation of biofilms after 2, 6, and 10 days of incubation in 35 mm Petri dishes was evaluated by Live/Dead staining (Life Technologies, Carlsbad, CA, USA) and Fluorescence Microscopy analysis. The Live/Dead assay kit contains SYTO 9 and propidium iodide (PI) with the intention of evaluating the cell viability. The green fluorescence shows the presence of live cells while the red fluorescence shows the presence of dead or damaged cells. The samples were analyzed by Fluorescence Leica 4000 DM Microscope (Leica Microsystems, Wetzlar, Germany). Broth cultures were plated on chocolate agar to ensure the absence of contaminating bacteria. All experiments were performed at room temperature, and each Petri dish was analyzed for no longer than 10 min. Results are the average of three independent experiments, containing triplicate samples.

The isolation of planktonic (pOMVs) and biofilm (bOMVs) vesicles was performed as previously reported^34^ after 2, 6, and 10 days of incubation. The weight of the pOMVs and bOMVs was measured and then used for further analysis. *Helicobacter pylori* cellular pellets were also collected, at each time point, from the planktonic and biofilm phenotypes as well as from overnight broth cultures to be used as controls.

### 2.4. Preparation of the whole cell extract (WCE) and OMVs lysates

Pellets of pOMVs, bOMVs, and WCEs were completely dissolved in Laemmli loading buffer containing SDS (1% final concentration). Samples subjected to protonography were dissolved in loading buffer without 2-mercaptoethanol. Each sample was normalized measuring the protein concentration using the Bradford assay.

### 2.5. SDS-PAGE and electroblotting

Sodium dodecyl sulfate (SDS)-polyacrylamide gel electrophoresis (PAGE) of the *H. pylori* lysates was performed using 12% gels as described previously^38^. Gel was stained with Coomassie blue. Blotting from gel onto an immobilion-P membrane was performed as described by Matsudaira et al^39^.

### 2.6. Protonography

The wells of 12% SDS-gel were loaded with the lysates of pOMVs, bOMVs, and WCEs prepared as aforementioned. A total of 30 µg of protein per well was loaded without 2-mercaptoethanol and without boiling the sample in order to avoid protein denaturation. Commercial bovine CA purchased from Sigma was used as positive control. The gel was run at 150 V until the dye front ran off the gel^40^. Following the electrophoresis, the 12% SDS-gel was subject to protonography to detect the OMVs or WCEs hydratase activity on the gel as described by Del Prete et al^41^^,^^42^. Briefly, the gel was removed from glass plates and soaked in 2.5% Triton X-100 for 1 h on a shaker and then twice in 100 mM Tris, pH 8.2 containing 10% isopropanol for 10 min. Subsequently, the gel was incubated in 0.1% bromothymol blue in 100 mM Tris, pH 8.2 for 30 min and then immersed in CO_2_-saturated ddH_2_O to visualize the hydratase activity of the enzyme. The assay was performed at room temperature, and the CO_2_-saturated solution was prepared by bubbling CO_2_ into 200 ml distilled water for approximately 3 h. The local decrease in pH due to the presence of CA activity was evidenced by the formation of yellow bands due to the change of the indicator color from blue (alkaline pH) to yellow (acidic pH).

### 2.7. Sample preparation for mass spectrometry analysis

The gel bands were treated as previously reported^41^^,^^42^. Briefly after washing the bands twice with a 25 mM solution of NH_4_HCO_3_ in 50% acetonitrile (washing buffer), the proteins were reduced with 10 mM dithiothreitol (DTT) (45 min, 56 °C) and alkylated with 55 mM iodoacetamide (IAA) (30 min, RT, in the dark). After another washing step, the bands were dried by adding 100% ACN for a couple of minutes, rehydrated for 30 min at 4 °C using an appropriate volume of trypsin solution (Promega; Madison, WI, USA) at 3 ng/μL in 50 mM NH_4_HCO_3_ and incubated at 37 °C overnight. The reaction was quenched by adding 10% trifluoroacetic acid (TFA). Each digested band was analyzed by LC-MS/MS using a Proxeon EASY-nLCII (Thermo Fisher Scientific, Milan, Italy) chromatographic system coupled to a Maxis HD UHR-TOF (Bruker Daltonics GmbH, Bremen, Germany) mass spectrometer as routinely performed in our laboratory^43^. 10 μL of sample was loaded on the EASY-Column TM C18 trapping column (2 cm L., 100 µm I.D, 5 µm ps, Thermo Fisher Scientific, Waltham, MA USA), and subsequently separated on an Acclaim PepMap100 C18 (75 µm I.D., 25 cm L, 5 µm ps, Thermo Fisher Scientific, Waltham, MA USA) nanoscale chromatographic column. The flow rate was set to 300 nL/min and the gradient was from 2 to 10% of B in 30′ followed by 10 to 18% in 20′, from 18 to 26% in 10′, from 26 to 50% in 15′ and from 50% to 90 in 5′ (total run time 90′). Mobile phase A was 0.1% formic acid in H_2_O and mobile phase B was 0.1% formic acid in acetonitrile. The mass spectrometer was equipped with a nanoESI spray source and operated in data-dependent acquisition mode (DDA), using N_2_ as the collision gas for CID fragmentation. Precursors in the range 350–2200 *m/z* (excluding 1220.0–1224.5 *m/z*) with a preferred charge state +2 to +5 and absolute intensity above 3137 counts were selected for fragmentation in a fixed cycle time of 3 s. After two spectra, the precursors were actively excluded from selection for 30 s. Isolation width and collision energy for MS/MS fragmentation were set according to the mass and charge state of the precursor ions (from 2+ to 5+ and from 21 eV to 55 eV). In-source reference lock mass (1221.9906 *m/z*) was acquired online throughout the runs. The raw data were processed using PEAKS Studio v7.5 software (Bioinformatic Solutions Inc, Waterloo, Canada) using the function “correct precursor only” as already reported^44^. Briefly, the mass lists were searched against a custom database containing *H. pylori* NCTC11637 proteins and a list of common contaminants (June 2017; 1880 entries) using 10 ppm and 0.05 Da as the highest error tolerances for parent and fragment ions, respectively. Carbamidomethylation of cysteines as fixed modification and oxidation of methionines and deamidation of asparagine and glutamine as variable modifications were selected allowing 2 missed cleavages. FDR at PSM level was set to 0.1% corresponding to an FDR at protein level around 1%.

## 3. Results and discussion

### 3.1. Characterization of the biofilm over time

**Figure 1.**
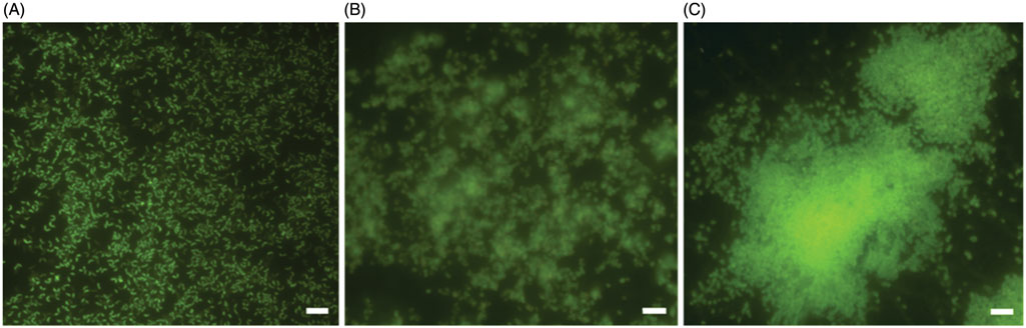
Representative images of fluorescence microscopy of *H. pylori* NCTC11637 biofilm development over time. The biofilms were stained with Live/Dead kit and visualized after 2 days (A); 6 days (B) and 10 days (C) of incubation. Scale bar =5 μm.

**Figure 2.**
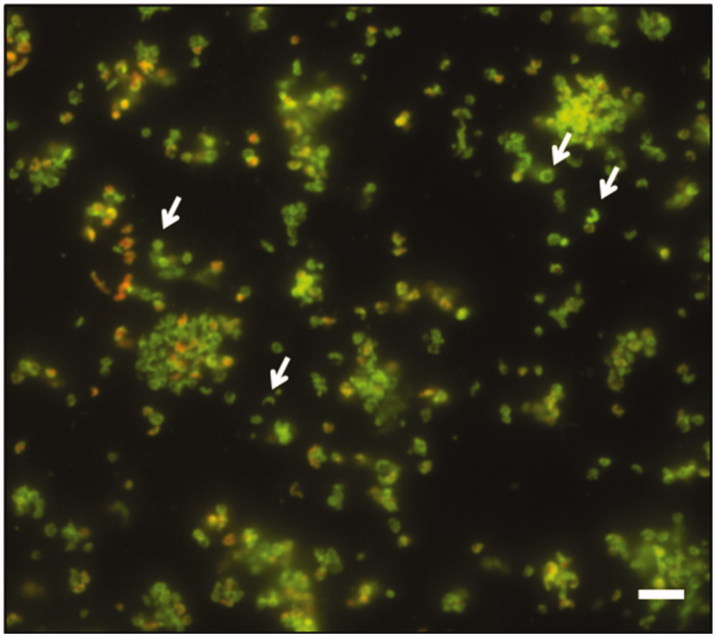
Representative image of fluorescence microscopy of *H. pylori* NCTC11637 biofilm after 10 days of incubation. The white arrows indicate the coccoid, donut and U-shape morphologies. The biofilm was stained with Live/Dead kit. Scale bar =5 μm.

The formation of biofilm over time was evaluated by live/dead staining and fluorescence microscopy analysis. All *H. pylori* strains developed a biofilm already after 2 days of incubation (Figure 1(A)), confirming what previously demonstrated^34^. Many single cells were attached to the polystyrene surface. The biofilms became more structured and organized (Figure 1(B)) up to assume a towers-like conformation after 10 days of incubation (Figure 1(C)). In fact, large cell aggregates in towers-like structures heterogeneously dispersed among voids with a few bacteria juxtaposed were visible after 10 days of incubation (Figure 1(C)). Furthermore, biofilms were characterized by a greater number of living cells (green) at each time point, while the number of dead cells (red) was negligible. The predominant cellular morphology after 2 days of incubation was the bacillary, replaced by coccoid, donut, and U-shape cellular morphologies at 6 and 10 days of incubation (Figure 2).

### 3.2. Identification of the hydratase activity in pOMVs, bOMVs, and WCEs

**Figure 3.**
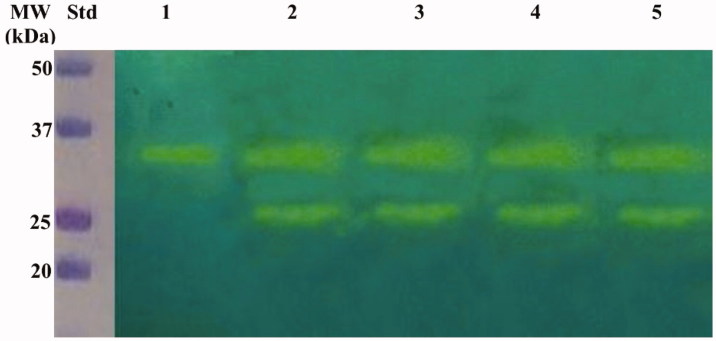
Hydratase activity on the polyacrylamide gel of WCEs obtained lysing the four *H. pylori* strains. The yellow band corresponds to the hydratase activity position on the gel. Lane STD, molecular markers; Lane 1: commercial bovine CA; Lane 2; *Hp* 190; Lane 3: *Hp* F1; Lane 4: *Hp* F4; Lane 5: *Hp* NCTC.

**Figure 4.**
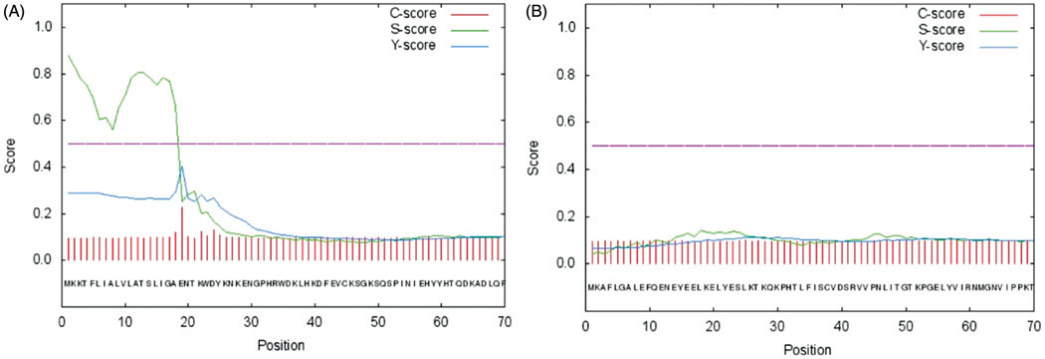
SignalP graphical output showing the three different scores C, S and Y, for the first 70 positions in typical α- and β-CAs encoded by the *H. pylori* genome. (A) α-CA from *H. pylori*; (B) β-CA from *H. pylori*. The program recognized the presence of a signal peptide at the N-terminal of the α-CA amino acid sequences, but did not detect any signal peptide in the β-CA. Legend: X-axis, amino acid position; C-score, raw cleavage site score; S-score, signal peptide score; Y-score, combined cleavage site score.

**Figure 5.**
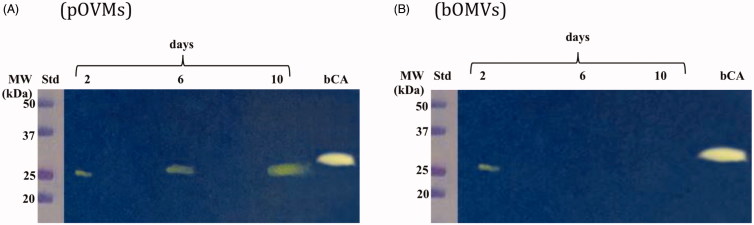
Hydratase activity on the polyacrylamide gel of OMVs produced by planktonic and biofilm phenotypes of *Hp* 190. The yellow band corresponds to the hydratase activity position on the gel. (A) Lysates of pOMVs; (B) lysates of bOMVs; Std, molecular markers; days, pOMVs or bOMVs after 2, 6 and 100 days; bCA, commercial bovine CA used as positive control.

The hydratase activity of WCEs and OMVs produced by *H. pylori* planktonic and biofilm phenotypes was investigated using protonography. WCEs and lysates prepared from pOMVs and bOMVs were loaded at 30 µg/well on the polyacrylamide gel. The protonography is a technique based on monitoring the pH variation in the gel due to the CA-catalyzed conversion of CO_2_ to bicarbonate and protons. The production of ions (H^+^) during the CO_2_ hydration reaction can be visualized as a yellow band on the gel. As expected, the WCEs obtained by the overnight growth of all the strains of interest evidenced two yellow bands with a molecular weight ranging from 26.0 to 28.0 kDa (Figure 3) when analyzed by protonography. As described in the literature, the genome of *H. pylori* encodes for α- and β-CAs, respectively, with a theoretical molecular mass of 28.35 and 25.65 kDa. The commercial bovine CA (bCA, M.W.= 28 kDa) was used as positive control. It is noteworthy that after removing SDS from the gel for developing the protonogram (see section 2.6), the metalloenzyme is able to correctly refold and generate its active form. This characteristic is also found in other CA classes belonging to prokaryotic and eukaryotic organisms. As reported in the literature, the bacterial α-CAs are secreted metalloenzymes characterized by a signal peptide (18–24 amino acids) at the N-terminus of the protein, which allows the molecule translocation across the cytoplasmic membrane and its localization into the bacterial periplasmic space^5^. In fact, the program SignalP 4.1 (http://www.cbs.dtu.dk/services/SignalP/)^45^, a program able to identify the signal peptide at the N-terminus of the protein, it is readily apparent that the α-CA encoded by the genome of *H. pylori* showed a signal peptide of 18 amino acid residues at the N-terminus, while it is missed in the bacterial β-CA (Figure 4). The signal peptide increases the molecular weight of the bacterial α-CA of about 2.0 kDa. The protonograms of Figure 5 were obtained using the lysates of the p and bOMVs produced after 2, 6, and 10 days by the bacterial strain *Hp* 190. Protonograms of the p- and bOMVs produced by *Hp* F1, *Hp* F4, and *Hp* NCTC have not been shown since they were very similar to the protonograms of Figure 5. Diversely from WCEs (Figure 3), the lysates of the p- and bOMVs originated by *Hp* 190 showed only a single yellow band with a molecular weight of approximately 26 kDa (Figure 5). Interestingly, the pOMVs hydratase activity increased over time, while the hydratase activity of the bOMVs disappeared after 2 days (Figure 5(A,B)). A possible explanation for this phenomenon is the release of the enzyme into the medium with the loss of activity in the bOMVs, as demonstrated by the detection of the activity in the liquid medium recovered by centrifugation at different days (data not shown). The identification of the hydratase activity in the p- and bOMVs is very intriguing because it could be strictly correlated to the extracellular DNA (eDNA) release. In fact, the eDNA is a component of the biofilm EPS matrix of many microorganisms and in *H. pylori* the eDNA is associated with OMVs produced in both the planktonic and biofilm phenotypes^34^. Rose and Bermudez showed that bicarbonate positively influences the release of eDNA in nontuberculous mycobacteria independently of pH^46^ and induces the eDNA release in *Mycobacterium avium*. The eDNA is involved in several mechanisms such as DNA damage repair, horizontal gene transfer (HGT) and as a source of nutrients^47^. Since the bacterial OMVs are characterized by proteins of the bacterial periplasmic space as described in the literature^48^, it has been supposed that the hydratase activity identified in the p- and bOMVs is due to the periplasmic bacterial α-CA, which is deprived of the signal peptide at the N-terminus because of its translocation in the periplasmic space. To validate our hypothesis, the bands corresponding to the molecular weight of 28 kDa coming from the WCEs (Figure 3) and 26 kDa detected in the p- and bOMV lysates (Figure 5) were electroblotted from the gel onto an immobilion-P membrane, excised from the membrane and subjected to mass spectrometry analysis.

### 3.3 Identification of proteins in WCE end pOMV lysates by mass spectrometry analysis

**Figure 6.**
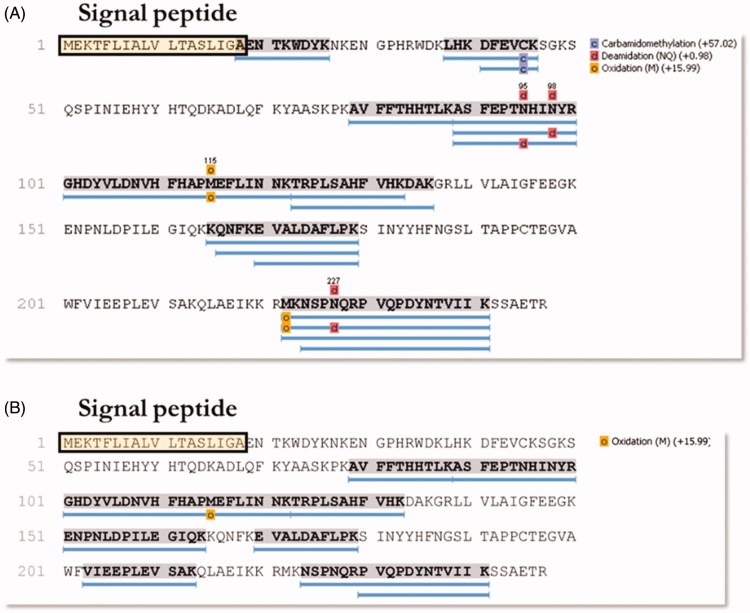
Schematic representation of the sequence coverage of the identified CAs in WCE (A) and pOMVs (B) from *H. pylori*. The signal peptide is highlighted on both panels. The overlap between the last AA of the signal peptide and the first AA of the identified sequence AENTKWDYK can be noted. The lists of all the identified proteins are provided as Supporting Information.

The 28 kDa and 26 kDa bands coming from the WCE fractioning SDS-Page were subjected to mass spectrometry analysis. 142 ± 4 proteins (FDR at the protein level =1.8%) were identified in the gel band at 28 kDa, while 163 ± 18 proteins (FDR at the protein level =2.9%) in the 26 kDa gel band. Among all the identified proteins, the α-CA (UniProt Accession number = I0ZDN6) was identified only in the band at 28 kDa, with 13 unique peptides and covering 45% of the sequence (Figure 6(A)). Excitingly, the mass spectrometry analysis of the 26 kDa band from enriched OMVs collected after culturing *H. pylori* in planktonic status (Figure 6(A)) generated 108 ± 18 proteins (FDR at the protein level =2.5%) and provided an α-CA, among the identified proteins, with 9 unique peptides and covering 44% of the sequence (Figure 6(B)). Similar results were obtained using the OMVs from the *H. pylori* biofilm phenotype (data not shown). It is worth noting a substantial difference in the coverage of the signal peptide region of α-CAs identified in WCE and pOMVs (Figure 6). The amino acid sequence of the peptide AENTKWDYK overlapping with the signal peptide (Figure 6, boxed amino acids) was only identified in the gel band at 28 kDa from WCE. Thus, these results support our previous hypothesis, which attributed OMVs hydratase activity to the *H. pylori* periplasmic α-CA characterized by the absence of the signal peptide.

## 4. Conclusion

Bacteria encode for CA belonging to the α-, β-, and γ-classes. These metalloenzymes contain zinc ion (Zn^2+^) in their active site, coordinated by three histidine residues in the α- and β-CAs or by two Cys and one His residues in the γ-CAs, with the fourth ligand being a water molecule/hydroxide ion. The genome of *H. pylori* encodes only for two different classes, the α- and β-CAs, which have a pivotal role for the acid acclimation of the microorganism within the human stomach. In this study using the protonography, a technique selective for the detection of CAs, it has been identified for the first time a hydratase activity in the OMVs generated by four strains of *H. pylori*. The protonograms showed that the hydratase activity had an expression level higher in the pOMVs than in the bOMVs. Interestingly, the pOMVs hydratase activity increased over time, while the hydratase activity of the bOMVs disappeared after 2 days because the enzyme was released into the medium. The mass spectrometry analysis carried out on the pOMV and bOMV lysates showed that the hydratase activity was due to the periplasmic bacterial α-CA, indicated with the acronym hpαCA. Further studies will be necessary to elucidate the role or the fate of α-CA delivered by OMVs in planktonic and biofilm phenotypes as well as a possible involvement of α-CA in eDNA release as previously demonstrated for nontuberculous mycobacteria^44^. However, these results shed new light in the field of the strategies used to combat the microbial infections. The inhibition of the periplasmic α-CA might block the colonization, survival, and pathogenicity merely interfering/destabilizing the production of the OMVs of the Gram-negative bacteria.

## Supplementary Material

Table_S3_list_of_IDs_WCE_28kDa_for_prod.xlsx

Table_S2_list_of_IDs_WCE_26kDa_for_prod.xlsx

Table_S1_list_of_IDs_pMVs_26kDa_for_prod.xlsx

Supplemental_Table_s_legend.docx

## References

[1] Capasso C, Supuran CT Bacterial, fungal and protozoan carbonic anhydrases as drug targets. Expert Opin Ther Targets 2015;19:1689–704. 26235676 10.1517/14728222.2015.1067685

[2] Del Prete S, Vullo D, di Fonzo P, et al. Comparison of the anion inhibition profiles of the beta- and gamma-carbonic anhydrases from the pathogenic bacterium *Burkholderia pseudomallei*. Bioorg Med Chem 2017;25:2010–5. 28238511 10.1016/j.bmc.2017.02.032

[3] Del Prete S, Vullo D, Di Fonzo P, et al. Anion inhibition profiles of the gamma-carbonic anhydrase from the pathogenic bacterium *Burkholderia pseudomallei* responsible of melioidosis and highly drug resistant to common antibiotics. Bioorg Med Chem 2017;25:575–80. 27914949 10.1016/j.bmc.2016.11.021

[4] Supuran CT, Capasso C Carbonic anhydrase from *Porphyromonas gingivalis* as a drug target. Pathogens 2017;6:30–42. 10.3390/pathogens6030030 PMC5617987 28714894

[5] Supuran CT, Capasso C New light on bacterial carbonic anhydrases phylogeny based on the analysis of signal peptide sequences. J Enzyme Inhib Med Chem 2016;31:1254–60. 27353388 10.1080/14756366.2016.1201479

[6] Capasso C, Supuran CT An overview of the alpha-, beta- and gamma-carbonic anhydrases from Bacteria: can bacterial carbonic anhydrases shed new light on evolution of bacteria?. J Enzyme Inhib Med Chem 2015;30:325–32. 24766661 10.3109/14756366.2014.910202

[7] Supuran CT, Capasso C An overview of the bacterial carbonic anhydrases. Metabolites 2017;7:56–74. 10.3390/metabo7040056 PMC5746736 29137134

[8] Berrino E, Bozdag M, Del Prete S, et al. Inhibition of alpha-, beta-, gamma-, and delta-carbonic anhydrases from bacteria and diatoms with N'-aryl-N-hydroxy-ureas. J Enzyme Inhib Med Chem 2018;33:1194–8. 30044657 10.1080/14756366.2018.1490733 PMC6060382

[9] Supuran CT, Capasso C Biomedical applications of prokaryotic carbonic anhydrases. Expert Opin Ther Pat 2018;28:745–754. 29973089 10.1080/13543776.2018.1497161

[10] Capasso C, Supuran CT Inhibition of bacterial carbonic anhydrases as a novel approach to escape drug resistance. Curr Top Med Chem 2017;17:1237–48. 28049405 10.2174/1568026617666170104101058

[11] Del Prete S, Vullo D, De Luca V, et al. Anion inhibition profiles of alpha-, beta- and gamma-carbonic anhydrases from the pathogenic bacterium *Vibrio cholerae*. Bioorg Med Chem 2016;24:3413–7. 27283786 10.1016/j.bmc.2016.05.029

[12] Capasso C, Supuran CT An overview of the selectivity and efficiency of the bacterial carbonic anhydrase inhibitors. Curr Med Chem 2015;22:2130–9. 25312213 10.2174/0929867321666141012174921

[13] Capasso C, Supuran CT Anti-infective carbonic anhydrase inhibitors: a patent and literature review. Expert Opin Ther Pat 2013;23:693–704. 23488877 10.1517/13543776.2013.778245

[14] Backert S, Neddermann M, Maubach G, et al. Pathogenesis of *Helicobacter pylori* infection. Helicobacter 2016;21:19–25. 27531534 10.1111/hel.12335

[15] Cellini L, Grande R, Artese L, et al. Detection of *Helicobacter pylori* in saliva and esophagus. New Microbiol 2010;33:351–7. 21213594

[16] Wen Y, Feng J, Scott DR, et al. The HP0165-HP0166 two-component system (ArsRS) regulates acid-induced expression of HP1186 alpha-carbonic anhydrase in *Helicobacter pylori* by activating the pH-dependent promoter. J Bacteriol 2007;189:2426–34. 17220228 10.1128/JB.01492-06 PMC1899393

[17] Sachs G, Kraut JA, Wen Y, et al. Urea transport in bacteria: acid acclimation by gastric *Helicobacter* spp. J Membr Biol 2006;212:71–82. 17264989 10.1007/s00232-006-0867-7

[18] Bury-Mone S, Mendz GL, Ball GE, et al. Roles of alpha and beta carbonic anhydrases of *Helicobacter pylori* in the urease-dependent response to acidity and in colonization of the murine gastric mucosa. Infect Immun 2008;76:497–509. 18025096 10.1128/IAI.00993-07 PMC2223474

[19] Nils StÃ¤hler F, Ganter L, Lederer K, et al. Mutational analysis of the *Helicobacter pylori* carbonic anhydrases. FEMS Immunol Med Microbiol 2005;44:183–9. 15866214 10.1016/j.femsim.2004.10.021

[20] Tsikas D, Hanff E, Brunner G *Helicobacter pylori*, its urease and carbonic anhydrases, and macrophage nitric oxide synthase. Trends Microbiol 2017;25:601–2. 28579469 10.1016/j.tim.2017.05.002

[21] Maresca A, Vullo D, Scozzafava A, et al. Inhibition of the alpha- and beta-carbonic anhydrases from the gastric pathogen *Helycobacter pylori* with anions. J Enzyme Inhib Med Chem 2013;28:388–91. 22299578 10.3109/14756366.2011.649268

[22] Modak JK, Liu YC, Supuran CT, et al. Structure–activity relationship for sulfonamide inhibition of *Helicobacter pylori* alpha-carbonic anhydrase. J Med Chem 2016;59:11098–109. 28002963 10.1021/acs.jmedchem.6b01333

[23] Morishita S, Nishimori I, Minakuchi T, et al. Cloning, polymorphism, and inhibition of beta-carbonic anhydrase of *Helicobacter pylori*. J Gastroenterol 2008;43:849–57. 19012038 10.1007/s00535-008-2240-3

[24] Nishimori I, Minakuchi T, Kohsaki T, et al. Carbonic anhydrase inhibitors: the beta-carbonic anhydrase from *Helicobacter pylori* is a new target for sulfonamide and sulfamate inhibitors. Bioorg Med Chem Lett 2007;17:3585–94. 17482815 10.1016/j.bmcl.2007.04.063

[25] Nishimori I, Minakuchi T, Morimoto K, et al. Carbonic anhydrase inhibitors: DNA cloning and inhibition studies of the alpha-carbonic anhydrase from *Helicobacter pylori*, a new target for developing sulfonamide and sulfamate gastric drugs. J Med Chem 2006;49:2117–26. 16539401 10.1021/jm0512600

[26] Nishimori I, Onishi S, Takeuchi H, et al. The alpha and beta classes carbonic anhydrases from *Helicobacter pylori* as novel drug targets. Curr Pharm Des 2008;14:622–30. 18336307 10.2174/138161208783877875

[27] Cellini L, Grande R, Traini T, et al. Biofilm formation and modulation of luxS and rpoD expression by *Helicobacter pylori*. Biofilms 2005;2:119–27.

[28] Grande R, Di Campli E, Di Bartolomeo S, et al. *Helicobacter pylori* biofilm: a protective environment for bacterial recombination. J Appl Microbiol 2012;113:669–76. 22639839 10.1111/j.1365-2672.2012.05351.x

[29] Wilkins M, Hall-Stoodley L, Allan RN, et al. New approaches to the treatment of biofilm-related infections. J Infect 2014;69: S47–S52. 25240819 10.1016/j.jinf.2014.07.014

[30] Grande R, Di Giulio M, Bessa LJ, et al. Extracellular DNA in *Helicobacter pylori* biofilm: a backstairs rumour. J Appl Microbiol 2011;110:490–8. 21143715 10.1111/j.1365-2672.2010.04911.x

[31] Hathroubi S, Servetas SL, Windham I, et al. *Helicobacter pylori* biofilm formation and its potential role in pathogenesis. Microbiol Mol Biol Rev 2018;82:1–15. 10.1128/MMBR.00001-18 PMC5968456 29743338

[32] Kulp A, Kuehn MJ Biological functions and biogenesis of secreted bacterial outer membrane vesicles. Annu Rev Microbiol 2010;64:163–84. 20825345 10.1146/annurev.micro.091208.073413 PMC3525469

[33] Orench-Rivera N, Kuehn MJ Environmentally controlled bacterial vesicle-mediated export. Cell Microbiol 2016;18:1525–36. 27673272 10.1111/cmi.12676 PMC5308445

[34] Grande R, Di Marcantonio MC, Robuffo I, et al. *Helicobacter pylori* ATCC 43629/NCTC 11639 outer membrane vesicles (OMVs) from biofilm and planktonic phase associated with extracellular DNA (eDNA. Front Microbiol 2015;6:1369. 26733944 10.3389/fmicb.2015.01369 PMC4679919

[35] Yonezawa H, Osaki T, Kurata S, et al. Outer membrane vesicles *of Helicobacter pylori* TK1402 are involved in biofilm formation. BMC Microbiol 2009;9:197. 19751530 10.1186/1471-2180-9-197 PMC2749055

[36] Snider CA, Voss BJ, McDonald WH, et al. Growth phase-dependent composition of the *Helicobacter pylori* exoproteome. J Proteomics 2016;130:94–107. 26363098 10.1016/j.jprot.2015.08.025 PMC4640983

[37] Sisto F, Scaltrito MM, Masia C, et al. Corrigendum to 'In vitro activity of artemisone and artemisinin derivatives against extracellular and intracellular *Helicobacter pylori*' [International Journal of Antimicrobial Agents 48/1 (2016) 101-105]. Int J Antimicrob Agents 2018;52:528–532. DOI: 10.1016/j.ijantimicag.2018.08.006. 27216383

[38] Laemmli UK Cleavage of structural proteins during the assembly of the head of bacteriophage T4. Nature 1970;227:680–5. 5432063 10.1038/227680a0

[39] Matsudaira P Sequence from picomole quantities of proteins electroblotted onto polyvinylidene difluoride membranes. J Biol Chem 1987;262:10035–8. 3611052

[40] De Luca V, Del Prete S, Supuran CT, et al. Protonography, a new technique for the analysis of carbonic anhydrase activity. J Enzyme Inhib Med Chem 2015;30:277–82. 25007132 10.3109/14756366.2014.917085

[41] Del Prete S, De Luca V, Iandolo E, et al. Protonography, a powerful tool for analyzing the activity and the oligomeric state of the gamma-carbonic anhydrase identified in the genome of *Porphyromonas gingivalis*. Bioorg Med Chem 2015;23:3747–50. 25910585 10.1016/j.bmc.2015.03.080

[42] Del Prete S, De Luca V, Supuran CT, et al. Protonography, a technique applicable for the analysis of eta-carbonic anhydrase activity. J Enzyme Inhib Med Chem 2015;30:920–4. 25676328 10.3109/14756366.2014.990963

[43] Alberio T, Pieroni L, Ronci M, et al. Toward the standardization of mitochondrial proteomics: the italian mitochondrial human proteome project initiative. J Proteome Res 2017;16:4319–29. 28828861 10.1021/acs.jproteome.7b00350

[44] Ronci M, Leporini L, Felaco P, et al. Proteomic characterization of a new asymmetric cellulose triacetate membrane for hemodialysis. Proteomics Clin Appl 2018;e1700140 DOI: 10.1002/prca.201700140. 29808585

[45] Petersen TN, Brunak S, von Heijne G, et al. SignalP 4.0: discriminating signal peptides from transmembrane regions. Nat Methods 2011;8:785–6. 21959131 10.1038/nmeth.1701

[46] Rose SJ, Bermudez LE Identification of bicarbonate as a trigger and genes involved with extracellular DNA export in mycobacterial biofilms. MBio 2016;7:1–11. 10.1128/mBio.01597-16 PMC5142616 27923918

[47] Ibanez de Aldecoa AL, Zafra O, Gonzalez-Pastor JE Mechanisms and regulation of extracellular DNA release and its biological roles in microbial communities. Front Microbiol 2017;8:1390. 28798731 10.3389/fmicb.2017.01390 PMC5527159

[48] Beveridge TJ Structures of gram-negative cell walls and their derived membrane vesicles. J Bacteriol 1999;181:4725–33. 10438737 10.1128/jb.181.16.4725-4733.1999 PMC93954

