# Supplementary material for: Identification and characterization of the α-CA in the outer membrane vesicles produced by Helicobacter pylori
Source: J Enzyme Inhib Med Chem. 2019 Jan 7;34(1):189–95. doi: 10.1080/14756366.2018.1539716 (PMC6327981; doi:10.1080/14756366.2018.1539716)
Supplement: Supplemental_Table_s_legend.docx [file IENZ_A_1539716_SM9077.docx]

**Table S1.** List of the identified proteins from the band at 26,0 kDa of pMVs as exported by the search engine. Every excel sheet contains the IDs of one technical replicate. The parameters provided in the table are: Accession number, Score of Identification (-10lgP), Protein Sequence Coverage (%), total number of matching peptides and number of unique peptides, number and type of PTMs, Average Mass and Description. Proteins are sorted by decreasing identification score. The proteins supposed to be contaminants are highlighted in orange while the carbonic anhydrase in green.

**Table S2.** List of the identified proteins from the band at 26,0 kDa of WCE as exported by the search engine.

**Table S3.** List of the identified proteins from the band at 28kDa of WCE as exported by the search engine.
